# Bridging the Gap: A Strategic Approach to Upscale Knowledge Among Diverse Healthcare Providers for Effective Tuberculosis Management in Gujarat, India

**DOI:** 10.7759/cureus.53255

**Published:** 2024-01-30

**Authors:** Harsh Shah, Jay Patel, Sandeep Rai, Anish Sinha, Deepak Saxena, Shikha Panchal

**Affiliations:** 1 Department of Public Health Sciences, Indian Institute of Public Health Gandhinagar, Gandhinagar, IND; 2 Department of Health and Family Welfare, Government of Gujarat, Gandhinagar, IND

**Keywords:** india, national tb elimination program, training, knowledge gaps, healthcare providers, tuberculosis

## Abstract

Introduction: Tuberculosis (TB) remains a global health challenge, particularly in low- and middle-income countries. Knowledge gaps among healthcare providers (HCPs) significantly impact TB management, hindering timely care-seeking and effective interventions.

Objective: The primary objective was to assess knowledge gaps among 3086 HCPs engaged in the National Tuberculosis Elimination Program (NTEP) implementation in Gujarat, India. The study provided a platform to develop and implement cadre-specific training modules to address identified knowledge deficiencies and enhance TB management.

Methodology: The study was conducted in two phases. Phase one was designed as a cross-sectional assessment to identify the knowledge gaps. Phase two involved the development of cadre-specific training modules based on identified deficiencies in the knowledge, crafted with collaboration from an expert panel. The training impact will be evaluated after completion of the training of all cadres through a comprehensive assessment.

Results: Out of 3086 assessed HCPs, 26% scored below the passing benchmark, revealing significant knowledge gaps. The variations were observed among and within the same cadres, with the accredited social health activists (ASHAs) and community health workers showing higher proficiency while pharmacists and medical officers showed lower proficiency. The cadre-specific training modules and training cascade were designed to address these gaps and improve TB-related knowledge and skills.

Conclusion: The study underscores the critical need for targeted interventions to address knowledge gaps among HCPs involved in TB control. The customized HCP-specific training programs are recommended to enhance knowledge, improve TB management, and contribute to national TB elimination goals.

## Introduction

In the complex network of global health, infectious diseases exert a profound impact on the well-being of societies, with tuberculosis (TB) emerging as a strong opponent. A leading cause of death and disability worldwide, TB exacts a particularly heavy toll on the health systems and economies of low- and middle-income countries (LMICs) [1].

*Mycobacterium tuberculosis*, the causative agent of TB, has persisted as the second leading cause of death from a single infectious agent. The gravity of the situation is evident in the 7.5 million newly diagnosed TB cases reported globally in 2022, with a striking 87% of these cases concentrated in 30 high-TB burden countries [2]. Notably, India bears a staggering burden, reporting 2.4 million cases annually, reinforcing the urgency of tailored and effective TB management strategies [3]. In response to the escalating TB crisis, the World Health Organization (WHO) has put forth the End TB Strategy, envisioning a reduction of 95% in TB deaths and 90% in incident cases by 2035. Aligning with this vision, the government of India has set an even more ambitious goal, aiming to eliminate TB from the country by 2025 [4]. However, achieving these targets necessitates a nuanced understanding of the challenges inherent in TB management, particularly as they are related to healthcare providers (HCPs).

The National Strategic Plan (NSP), encapsulated in the "Detect - Treat - Prevent - Build" pillars, aims for an annual tuberculosis incidence reduction of over 10-15%. Emphasizing staff training under the Build pillar, the NSP stresses the need for a proficient workforce within the program. To address the training demand for approximately two million HCPs directly or indirectly involved in healthcare and TB services, the NSP advocates a scalable system and the adoption of e-learning methodologies [4]. The success of TB control programs depends on timely care-seeking, accurate diagnosis, and prompt initiation of treatment. Yet, persistent delays in these crucial stages halt the desired outcomes of TB control efforts [5-7]. A pivotal aspect contributing to these challenges lies in the knowledge gaps among HCPs, manifesting in suboptimal care provision, late detection, poor treatment adherence, and the emergence of drug-resistant TB [8,9].

A three-phase intervention was designed to enhance the proficiency, precision, and patient-centered approach in TB management for HCPs by identifying particular areas of knowledge deficiency, developing tailored training materials, and imparting training. The current training methods in the health system follow a uniform approach and do not adequately consider the varying requirements of different groups within the healthcare system. It is crucial to identify precise areas of knowledge deficiency among HCPs and accordingly provide customized training modules to address these deficiencies; assessment of health workers needs to be carried out at regular intervals to assess the impact of training imparted among them [10-16]. The present study reported the outcomes of the first two phases, which included a description of the results from the knowledge assessment and the approach used to build the training modules.

## Materials and methods

Study design

This intervention study progressed through two distinct phases. The initial phase consisted of a questionnaire-based, cross-sectional, observational study that focused on the knowledge assessment of frontline HCPs. Subsequently, the second phase consisted of identifying critical knowledge gaps, which were then utilized to create cadre-specific training modules. The study was approved by the Institutional Ethical Committee of the Indian Institute of Public Health Gandhinagar, Gujarat (approval number: TRC/2020-21/18). Administrative permission was also secured from the Regional Deputy Director, Bhavnagar Regional Office, Health and Family Welfare Department (HFWD), Government of Gujarat, ensuring compliance with ethical standards and regulatory protocols.

Study settings

The study was conducted in Gujarat, a state in Western India. The state has a population of 72.7 million across 33 districts and eight municipal corporations [17]. The present intervention was conducted in the Bhavnagar region of Gujarat, covering five districts and two corporations, a total of eight units. The total duration of the intervention was two years, with the initial two phases lasting approximately nine months, from October 2022 to May 2023.

The NTEP is rigorously implemented across all districts of India. District-level monitoring is provided by dedicated District TB Centres, further divided into Tuberculosis Units (TUs) at the sub-district level. TUs are staffed with a team comprising a Medical Officer-Tuberculosis Control (MO-TC), Senior Treatment Supervisor (STS), and Senior TB Laboratory Supervisor (STLS), overseeing TB control activities. Diagnostic, treatment, and preventive interventions are executed through designated microscopy centres (DMCs) and peripheral health institutions (PHIs) HCPs.

Study population

The study population comprised a diverse group of HCPs (n=3086) actively engaged in the NTEP program within the selected units (districts and corporations), operating at and below the block level of government health facilities, including Taluka Health Officers (THO), Medical Officers (MO) at primary health centres (PHC), Ayush MO, Community Health Officers (CHO), pharmacists, STSs, STLSs, laboratory technicians (LTs), multi-purpose health worker (MPHWs), multi-purpose health supervisors (MPHS), TB health visitors (TBHVs), and accredited social health activists (ASHAs).

HCPs actively engaged in the NTEP and operating at and below the block level were included. Those who expressed unwillingness to participate, were engaged in other activities, or were absent during the assessment were excluded.

Phase I: knowledge assessment

**Figure 1:**
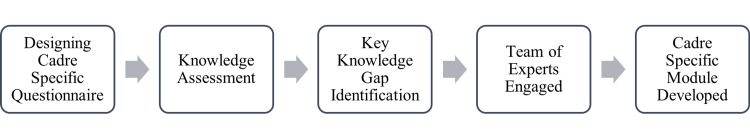
Activities for the initial two phases of the study intervention

In the initial phase, as per Figure 1, key stakeholders at the block level were meticulously identified due to their pivotal role in the delivery of TB services. Stakeholders play a crucial part in the effective implementation of the TB program.

Data Collection

Designing of the questionnaire: A comprehensive approach to data collection was employed to capture the depth of knowledge and expertise among HCPs. This involved the meticulous design and deployment of a cadre-specific knowledge assessment questionnaire. This tool underwent thorough pretesting conducted at least 10 times among participants such as THO, MO at PHC, Ayush MO, CHO, pharmacist, STS/STLS, and various community health workers. The validation processes included the assessment of 10 carefully formulated questions. The questions were strategically formulated to evaluate respondents' proficiency as per their roles and responsibilities across critical domains, including TB case screening, diagnosis, treatment, and programmatic actions.

Setting a robust benchmark for proficiency, the passing threshold was established at 60% of the total marks, ensuring a stringent evaluation of participants' grasp of TB-related knowledge in various areas of TB care. By utilizing the digitally assisted random-order question evaluation tool, the approach guaranteed the quality of data collected and enabled a nuanced analysis of the knowledge landscape among HCPs.

Deployment of questionnaire through Ni-kshay Setu application: To ensure efficiency, consistency, and standardized delivery, the data collection process was executed using an online questionnaire created on the knowledge assessment section of the Ni-kshay Setu application (https://nikshay-setu.in/) [18]. This NTEP-specific knowledge enhancement mHealth (mobile health) tool has been implemented in Gujarat by the State TB Cell. This innovative approach not only facilitated seamless data gathering but also leveraged technology to enhance the overall reliability and validity of the study. The application-based delivery mechanism provided a user-friendly interface for participants, streamlining their engagement with the questionnaire. By embracing technological advancements, we aimed to reduce biases associated with traditional data collection methods and foster a more dynamic and responsive research environment. The utilization of the Ni-kshay Setu application underscored our commitment to precision and reliability in data collection, aligning with the rigour expected in scientific research. This tool not only aided in collecting data efficiently but also streamlined the analysis process, making deployment and customization easier, and ensured the confidentiality of the questions.

Phase II: training content development

This pivotal stage was characterized by a meticulous focus on the systematic development of specialized training modules strategically tailored to bridge the identified knowledge gaps among HCPs (Figure 1). The primary objective was to coordinate a focused and efficient response to the issues indicated in the initial evaluation, with the ultimate goal of addressing the deficiencies in TB management.

The panel was constituted of a team of experts carefully curated from the region and facilitated by the State TB Cell. The panel, consisting of professionals with extensive experience in providing training and developing training modules for NTEP, had a crucial role in influencing the direction of our intervention. Their collective expertise spanned various facets of healthcare, including TB management, program implementation, and educational strategies. This panel created training content tailored to specific healthcare system cadres. This participatory approach guaranteed that the training resources were comprehensive and tailored to each cadre's TB management function. During the third phase of the intervention, these training resource materials will be utilized to provide training to the study population. After the training, the modules will be made available on a digital platform for the HCPs to access easily. This will allow them to take action based on their roles and responsibilities, addressing the challenges they face in the field. Their knowledge will be assessed again after the training is implemented.

Data analysis

The analysis of the study data employed descriptive statistics, average, quartile range, and standard deviation to summarize the scores obtained by HCPs in the knowledge assessment questionnaire. The score analysis was conducted based on cadres and areas of knowledge gaps. Unit, cadre, and knowledge-specific analyses were also conducted. This analytical approach was aimed at providing a granular understanding of the existing knowledge landscape, informing future interventions and improvements in the TB control program.

## Results

Phase 1: knowledge assessment

A total of 3086 HCPs actively engaged in the NTEP within the Bhavnagar region were included in the knowledge assessment. The results revealed noteworthy variations in the knowledge score of HCPs across different cadres within the NTEP. The passing benchmark for the assessment was set at 60% of the total marks. Among the assessed HCPs, 2282 (74%) demonstrated a strong grasp of TB-related knowledge, achieving a 60% or higher passing score. Conversely, 804 (26%) HCPs fell below the passing benchmark, shedding light on the existence of knowledge gaps within this pivotal workforce (Table 1).

**Table 1:** Knowledge assessment score as per various cadres MO: Medical Officer; ASHA: accredited social health activists; MPHW: multi-purpose health worker; MPHS: multi-purpose health supervisor; TB: tuberculosis

S. N	Cadres of healthcare providers	N	Score of 100%, n (%)	Score of 81-90% marks, n (%)	Score of 61-80% marks, n (%)	Score of <60% marks, n (%)
1	ASHA	1341	526 (39%)	336 (25%)	340 (25%)	139 (10%)
2	Community Health Officer/Ayush MO	739	137 (19%)	115 (16%)	174 (24%)	313 (42%)
3	MPHW and MPHS	554	163 (29%)	122 (22%)	155 (28%)	114 (21%)
4	Laboratory Technician	91	12 (13%)	15 (16%)	24 (26%)	40 (44%)
5	Pharmacist	160	1 (1%)	17 (11%)	38 (24%)	104 (65%)
6	Senior Treatment Supervisor	51	15 (29%)	11 (22%)	14 (27%)	11 (22%)
7	Senior TB Laboratory Supervisor	24	0 (0%)	3 (13%)	13 (54%)	8 (33%)
8	TB Health Visitor	12	4 (33%)	3 (25%)	3 (25%)	2 (17%)
9	MO and Taluka Health Officer	114	1 (1%)	3 (3%)	37 (32%)	73 (64%)
Total		3086	859 (28%)	625 (20%)	798 (26%)	804 (26%)

**Figure 2:**
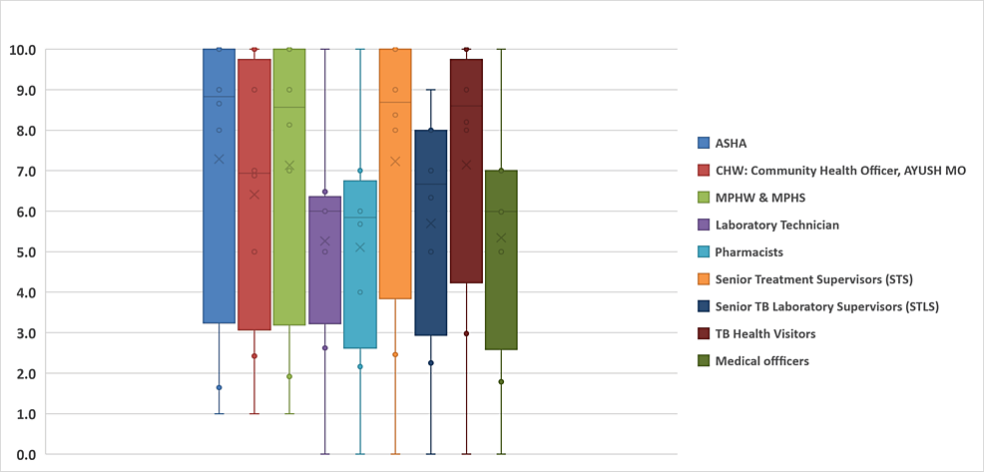
Knowledge score of various cadres under NTEP NTEP: National Tuberculosis Elimination Program; MO: Medical Officer; ASHA: accredited social health activists; MPHW: multi-purpose health worker; MPHS: multi-purpose health supervisor; TB: tuberculosis; CHW: community health worker

The detailed breakdown of results by cadre illuminates the nuances in knowledge levels among diverse healthcare roles within the NTEP (Figure 2). Notably, ASHA workers and CHO/Ayush MOs exhibited relatively higher performance, with 39% and 19% achieving scores of 100% each. In contrast, certain cadres such as pharmacists and MO/THO revealed a significant need for improvement, with 65% and 64% scoring below the passing benchmark, respectively.

As per Figure 2, the knowledge assessment results present a nuanced landscape, affirming a commendable overall proficiency among NTEP staff, with a mean score of 7.4 across all cadres. This suggests a robust understanding of TB prevention and treatment within the program.

However, variations emerged when scrutinizing individual cadres. The assessment of knowledge among different cadres focused on three main areas: Case Findings, TB Diagnosis and Treatment, and Programmatic Actions. Cadres were assessed according to their roles and carried responsibilities in these specific areas of the TB cascade. Notably, ASHAs (mean score 8.7), STSs (mean score 8.4), and TB health visitors (mean score 8.2) exhibited the highest mean scores, showcasing a commendable depth of knowledge. Conversely, laboratory technicians (mean score 6.5), MOs (mean score 6.0), and pharmacists (mean score 5.7) demonstrated lowest mean scores, indicating potential areas for improvement.

The standard deviation and quartile range values revealed an intriguing mix of high- and low-scoring individuals within each cadre. This implies that, even within groups with relatively lower mean scores, there were individuals demonstrating noteworthy proficiency. The dispersion in scores within cadres highlighted the diversity in knowledge levels as per their roles and responsibilities, suggesting a complex interplay of factors influencing individual performance. Examining the minimum and maximum scores within each cadre offered a comprehensive view of the range of knowledge among staff. For instance, it was observed that ASHAs with scores spanning from 1 to 10, and MOs, with scores ranging from 0 to 10, showcased a diverse spectrum of understanding.

Disparities in training, experience, and job responsibilities could contribute to these differences. For instance, cadres with higher mean scores may benefit from more extensive or targeted training initiatives, while those with lower mean scores may require tailored interventions to address specific knowledge gaps.

Phase 2: training content development

In response to the critical knowledge gaps identified through the comprehensive assessment of HCPs in the NTEP Program (Table 2), the second phase of our study moved towards a proactive intervention. Recognizing the unique needs of different cadres, cadre-specific training modules were meticulously developed to bridge the identified deficiencies and elevate the overall proficiency of HCPs.

**Table 2:** Categorisation and prioritisation of cadre-specific training areas +++ = 10% Unanswered or Wrong Answered; ++ = 11-20% Unanswered or Wrong Answered; + = 21-30% Unanswered or Wrong Answered

S. N	Cadres of healthcare providers	Areas of knowledge assessment		
		Case Findings	TB Diagnosis and Treatment	Program-related Actions
1	Accredited Social Health Activist	++	+++	++
2	Community Health Officer	++	+++	+
3	Multi-purpose Health Workers	+++	++	++
4	Laboratory Technicians	+++	++	++
5	Pharmacists	++	+	++
6	Senior Treatment Supervisors	+++	+++	++
7	Senior TB Laboratory Supervisors	+++	++	++
8	TB Health Visitors	+++	++	++
9	Medical Officers at Health Facilities	++	+++	+

**Figure 3:**
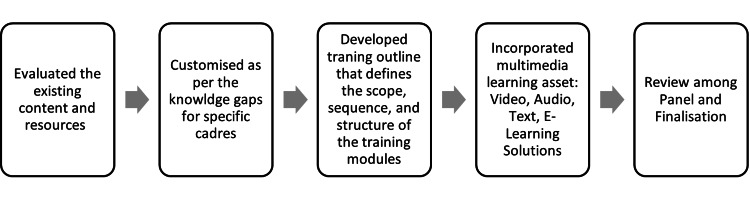
Workflow of developing cadre-specific modules

For HCPs such as CHOs and MOs at PHC, specialized training modules were specifically designed to accentuate program-related actions to enhance their knowledge. The modules for the other cadres were also carefully designed to address specific knowledge gaps and prioritize content that improved their understanding of program-related aspects. The ultimate goal was to empower HCPs with the necessary skills and insights for effective program implementation and TB case management based on their knowledge gaps. The development process involved a thorough review of the latest program guidelines to ensure alignment with current standards and best practices. The content was curated with meticulous attention to detail, encapsulating the most up-to-date information, strategies, and skills needed for managing TB cases in real-world situations. The second phase followed the critical steps in designing the content, as shown in Figure 3.

A key facet of the intervention strategy was the delivery of modular and on-the-job training by providing a dynamic platform for healthcare workers to integrate the acquired knowledge directly into their daily responsibilities. The three-phase intervention was directed to improve information retention and healthcare personnel's TB care by encouraging hands-on, practical learning.

## Discussion

The knowledge assessment outcomes underscore a critical concern, revealing that 26% of HCPs scored below the passing benchmark of 60%. This alarming finding emphasized the existence of knowledge gaps within a significant segment of the workforce responsible for TB control in the sample units of the Bhavnagar region of Gujarat, India. Notably, these results aligned with previous studies highlighting poor overall knowledge among HCPs regarding TB, including insufficient understanding of the disease's nature and diagnostic aspects [19].

The present study revealed a clear difference in knowledge levels among various groups, which supports earlier research suggesting that these disparities are due to inadequate availability of comprehensive training and continued education opportunities. The crucial role played by these HCPs in community outreach and education necessitates a robust foundation in early case detection and referral. The training programs delivered through accessible means, such as interactive workshops, on-the-job training, or e-learning platforms, could empower them with the skills and confidence to contribute effectively [20-23].

The current study adds evidence in advocating customized training approaches to address the specific knowledge gaps prevalent among HCPs involved in TB control. Customized training content, attuned to the unique needs of diverse cadres, emerges as a cornerstone for enhancing competence and ultimately improving TB management. A noteworthy study in 2020 reinforces this notion, showcasing that customized cadre-specific training can yield substantial improvements in HCP knowledge, consequently leading to enhanced patient outcomes [24]. According to the study by Hoffman et al., such interventions increase not only HCPs' knowledge but also patient outcomes [25].

The current study advocates for continuous training for healthcare providers through developing a training cascade to deliver orientation of newer program updates or new skills in terms of patient management. Simultaneously, monitoring and feedback mechanisms should be maintained to sustain the gains made through the intervention over time. This iterative process not only safeguards against knowledge erosion but also fosters a culture of continuous improvement within the healthcare delivery landscape [19,26-28].

The implications of our findings extend beyond the confines of the Bhavnagar region, holding relevance for the entire NTEP framework and analogous TB control programs across India. Addressing knowledge deficiencies through customized training content, developing a training cascade calendar, and leveraging digital technology (e-learning platforms like Ni-kshay Setu) emerges as a potent strategy. By equipping HCPs with specific knowledge and abilities, TB control programs can provide a measurable improvement in patient outcomes, decrease transmission rates, and lead to significant advancement toward the national objective of eliminating TB [20,21,29].

Strength and limitations

While the study offered valuable insights into addressing knowledge gaps among HCPs in TB control, it had limitations. The findings were specific to the Bhavnagar region in Gujarat, India, and cannot be universally applicable. The identified cadres and their training needs may differ in other regions, requiring context-specific adaptations and administrative support. The third phase of the intervention would generate more in-depth observations on the retention of knowledge, their feedback on training content, and the patient outcome status of the particular region. External factors like policy and administrative changes or infrastructure improvements were not explicitly considered, affecting the generalizability of findings.

## Conclusions

The present findings expose the critical need to look at the capacity-building component of NTEP from different perspectives. The knowledge and skills of HCPs under the "Build" pillar of the NSP should be enhanced with a restructuring of the existing conventional training approach. Therefore, the study suggests customized training programs, strengthened with digital knowledge management tools, as a potent strategy to empower HCPs. The comprehensive approach of training may strengthen the health system to deliver more effective TB management, propelling us closer to achieving the national TB elimination goals.
